# Responding to Appalachian Voices: Steps in Developing Substance-Use Recovery Ecosystems

**DOI:** 10.13023/jah.0203.10

**Published:** 2020-07-19

**Authors:** Bruce Behringer

**Affiliations:** behr320@hotmail.com

**Keywords:** Appalachia, drug abuse, opioids, recovery, rural communities, mortality rate, overdose

## Abstract

A description is presented of the four-step process used by the Appalachian Regional Commission to develop a new Recovery to Work initiative. The Commission identified, defined, and described issues facing individuals who complete substance abuse disorder treatment and who seek reentry into the workforce. Key elements were identified for resources and supports to develop and maintain community-based substance abuse recovery ecosystems. The steps included conceptualization, data collection, analysis, and review to formulate recommendations for program and policy development. The full process of development was accomplished in twelve months.

## BACKGROUND

Appalachian Regional Commission (ARC) officials noted continuing evidence and received continuous testimony about the negative impacts of the high prevalence of substance abuse on the region’s workforce participation rates and its overall economy. While the primary types of substances abused have morphed over time, both qualitative and quantitative findings point to a disparity between national and Appalachian substance-abuse statistics.^1^,^2^ Many federal and state agencies have taken up the charge to prevent substance misuse and to ensure that adequate and effective treatment services become available.

The Appalachian Regional Commission has taken a major role since 2000 in documenting substance-abuse disparities and drawing national attention and resources to the region through sponsoring conferences, supporting research studies, and funding small grant programs. An initial study in 2004 documented the nation’s first geographic region of disparities for health conditions and poor population-based outcomes.^3^ Further studies focused on mental health services shortages^4^ and substance-abuse outcomes.^5^–^7^ This regional presence and awareness has spawned interest in cooperative research and dissemination partnerships with national organizations including NORC at the University of Chicago,^8^ the National Association of Counties,^9^ and the Robert Wood Johnson Foundation.^10^ ARC has also engaged federal agencies including the Office of National Drug Control Policy, the Federal Office of Rural Health Policy, and the Substance Abuse Services and Mental Health Administration to bring additional attention and resources to the region.

One aspect of the regional substance-abuse issue, elevated through pervasive stories, concerns difficulties faced by people who complete substance-abuse treatment and who seek meaningful employment. Feedback provided by local and state officials indicated there is a significant number of people completing substance-abuse disorder (SUD) treatment and encountering problems with continuing recovery, being prepared for work, and finding jobs. An internal review found limited references in government reports or other published sources about this subject.

Appalachian Regional Commission Federal Co-Chair Tim Thomas identified interest among state economic development officials, regional businesses, and law enforcement in identifying how to assist people who had completed substance-abuse treatment through recovery to gain employment. In some communities and states, there seemed to be a large array of services, agreements, and structures in place that, if effectively organized, could form key elements of a recovery ecosystem. Other areas reported absence of services and providers. The Federal Co-Chair desired to engage state and local expertise to define challenges, identify promising approaches, and formulate the recommendations to inform future ARC actions in response to these issues.

Viewpoints and contributions of multiple sectors were needed, including the voices of people in recovery, organizations providing treatment and recovery services, workforce development agencies, and employers. In 2019 ARC determined to engage a community-based participatory process for defining critical elements of recovery ecosystems, assessing priorities, and planning effective approaches for a unique regional recovery-to-work initiative proposed for 2020.

## METHODS

The development of the recovery-to-work Initiative progressed over 12 months in four steps. In general these steps included (1) conceptualization of the recovery ecosystem model, (2) gathering ideas from the field that would help identify key elements of the model and existing related successful interventions, (3) review of ideas by a panel of experts leading to recommendations for action, and (4) the development of a range of ARC program interventions.

### Step 1: Developing the ARC Recovery Ecosystem Model

**Figure 1:**
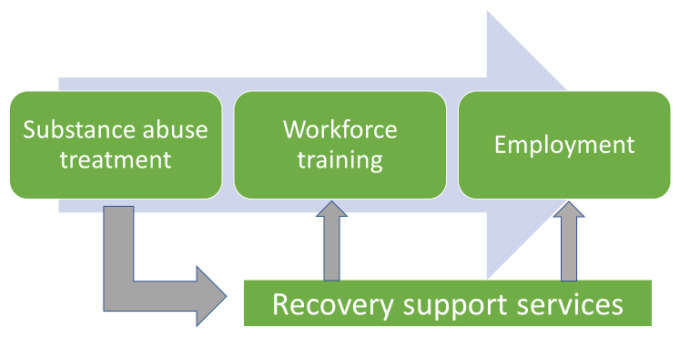
The ARC Recovery Ecosystem Model

The ARC staff conducted a planning meeting in October 2018 at which a graphic flow chart (Figure 1) was generated to define key elements of a Recovery Ecosystem Model (the Model). In conceptualizing a recovery-to-work initiative, ARC chose to focus attention on issues that follow an individual’s treatment for substance-abuse disorder with the long-term goal of workforce reentry and employment. Two intermediate steps were identified: workforce development services and continued recovery support services throughout the process. These steps recognized and required a multi-sector approach. No scalable existing recovery ecosystem program models had been identified, so ARC chose to gather input about types of services and linkages required for an effective model through a series of region-based listening sessions. Local ideas were solicited using Recovery Ecosystem Model framework. The combined regional input was then reviewed by an Appalachian Substance Abuse Advisory Council charged to develop recommendations for ARC action.

### Step 2: Recovery-to-Work Listening Sessions

Listening sessions were conducted at community colleges and a state park in the Appalachian regions of six states (Virginia, North Carolina, Alabama, Kentucky, Ohio, and West Virginia). ARC state liaisons and local development districts (LDDs) provided assistance in organizing meetings, supporting logistics, advertising, and recruiting speakers and participants. Single day sessions were organized with a 6-hour agenda. Listening sessions were conducted in the morning; public meetings were held in the afternoon. Five of six states followed this approach. One state conducted a single roundtable discussion. ARC identified a moderator to facilitate all meetings.^11^

The invitational listening session included short presentations from ARC staff about the purpose of the meeting, from a state official about substance-abuse and workforce issues, and from a local person in recovery telling about their personal journey. Small groups of participants then rotated around three flip charts that contained questions about each element of the Recovery Ecosystem Model. Facilitated discussion among all participants clarified and expanded ideas recorded on the flip charts. Average listening session attendance was 25 people. The advertised afternoon public meeting opened with comments from ARC followed by a panel of state and regional speakers who described local issues. One speaker represented each of the three elements of the Recovery Ecosystem Model: recovery support services, job training programs, and employers. A facilitated discussion encouraged participation to gather additional perspectives. Average public meeting attendance was 75 people.

Data were collected using three methods. Flip charts were used at listening sessions to record ideas. At the public meetings index cards were used to gather written responses to the statement, “The most important recommendation I would make to ARC regarding designing and planning initiatives to help adults with substance-abuse disorder secure meaningful employment following treatment is….” Field notes were taken throughout listening sessions and public meetings by ARC staff and the moderator. Separate reports were prepared for each state meeting based on the transcribed sources. Input from all six meetings was combined resulting in a large number of ideas and themes.

Ideas were categorized using the Recovery Ecosystem Model elements. Themes and subthemes were generated. A second sort was conducted to assign ideas into steps of a traditional planning pyramid (i.e., problem statements, goals for change, alternative strategies, effective practices/programs). The final report of the recovery-to-work regional meetings included tables summarizing themes and data using the Model and planning pyramid.

### Step 3: Appointment and Recommendations from the ARC Substance Abuse Advisory Council

The Appalachian Regional Commission created a Health Policy Advisory Council in 2000 with representatives drawn from each of the thirteen states with Appalachian counties. In 2019, this Council was reformed, renamed the ARC Substance Abuse Advisory Council, and appointed to focus on substance-abuse issues. Following consultation with ARC state liaisons, 24 people from the Appalachian regions of all 13 states received invitational letters of appointment in April 2019 from the ARC Federal Co-Chair. Membership was purposefully mixed. State and local governments, including LDD representatives, provided economic development viewpoints with updates on state policies and programming. Law enforcement contributed insights about legal and social issues. Community members, including recovery service and advocacy group representatives, brought the voices of those in recovery as well as their families and neighbors. Education and training organization representatives contributed experiences from outreach and operational adaptations that helped those in recovery obtain skills needed for employment. Employers reported on workforce trends and policies that facilitated or impinged on hiring and retaining those in recovery. Larger multicounty recovery service provider members shared programming experience. One-third of Council members attended one of the listening sessions.

The Council convened in two multi-day meetings in 2019, on May 15–16 in Knoxville, Tennessee, and July 16–17 in Washington DC. The Council was tasked with developing recommendations, achievable within ARC’s mission, to help individuals in recovery get the support services and training they need to maintain recovery and successfully reenter the workforce. Council deliberations included review of input from the listening sessions. Recommendations were formulated to define, build, and strengthen recovery ecosystems across Appalachia. The Council’s recommendations were considered by ARC at its Annual Summit in Asheville, North Carolina, in September 2019.

Appalachian Regional Commission staff provided background on the Recovery Ecosystem Model at the first meeting, described within the ARC mission of regional economic and workforce development. Council members who attended listening sessions shared insights about the tone and content from their state’s sessions. Several members with statewide substance-abuse responsibilities provided an overview of current federal programming and examples of state initiatives in recovery and workforce efforts.

Council members were assigned to small groups to review data from listening sessions and public meetings. Each group reviewed one element of the Recovery Ecosystem Model and presented findings for full Council discussion. Council members then joined a work group, one group for each Model element, to organize preliminary recommendations. These were formatted using steps of a planning pyramid, including problem statements, goals for change, broad strategic approaches, and program activities. This ensured that each solution (e.g., proposed programs) was linked to one or more strategies that emerged from stated problems and associated goals for change. Each work group presented drafts of multiple recommendations for full Council discussion by the end of Meeting 1. Work group volunteers convened via conference calls to improve their recommendations based on Council questions and feedback prior to Meeting 2.

At Meeting 2, work groups presented their redrafted recommendations for further Council discussion. Groups reconvened, finalized single-page recommendations on PowerPoint slides, and presented their products again for amendments and final wordsmithing. During this review, several gaps and overlaps were identified that led to new combinations and new recommendations. The full Council voted approval of a package of 14 recommendations with permissions given to members to submit final wording changes to the authoring work group.

Following this discussion, the facilitator led Council members to develop a set of “We Believe” statements that summarized the Council’s beliefs about the ARC recovery ecosystem approach and expected outcomes. These declarative sentences were used as a preface in the Council recommendations report.

The ARC Policy Group reviewed and approved the recommendations at its July meeting. Five of the 14 recommendations were suggested as priorities for initial action. The full Appalachian Regional Commission adopted the Policy Group’s report, including Council recommendations, at the September annual meeting and charged ARC staff with integrating the recommendations into action plans.

### Step 4: ARC Investment Strategy to Support Appalachian Recovery Ecosystems

While ARC had invested funds to address workforce participation and substance-abuse disorder (SUD) as separate challenges, no combined, comprehensive strategy had been established. Step 2, the listening sessions, and Step 3, the Advisory Council review, verified the importance of a local recovery ecosystem approach to address regional workforce reentry issues for people with SUD. ARC used the Council’s recommendations to inform future funding investments. A new $10 million federal budget appropriation was approved for Fiscal Year 2020 to support a recovery-to-work initiative in the Appalachian region. ARC also inserted a priority provision to strengthen substance-abuse responses in the large Partnerships for Opportunity and Workforce and Economic Revitalization (POWER) grant for communities and regions affected by job losses in coal mining, coal power plant operations, and coal-related supply chain industries. The involvement of states in the listening sessions and on the Council encouraged their engagement in and promotion of the new initiative. The new initiative fit into the historic ARC funding process, which relies on state development of packages of funding proposals for ARC review.^12^

## RESULTS

The four-step process demonstrated a proactive, regional approach for gathering and using community-level input to define problems, target programs, design appropriations, and form local partnerships to address the intersection of the region’s substance-abuse and economic development problems. Participants at state meetings and members of the Substance Abuse Advisory Council viewed the recovery-to-work focus as unique and badly needed. Seeking the “wisdom of the field” verified impacts of substance abuse on the region’s workforce and many local employment issues. Many state and local entities expressed support for the Recovery Ecosystem Model approach as a means to bring many interests together.

The Recovery Ecosystem Model was a successful framework for gathering and organizing ideas through the listening sessions and public meetings. The ideas were then used by the Council to develop recommendations. The recommendations were adopted by ARC to offer regionally responsive and targeted funding opportunities to address recovery-to-work challenges.

### Listening Session and Public Meeting Ideas

Almost 1000 ideas were generated through the six listening sessions and public meetings.^13^,^14^ These ideas were sorted into three Model elements categories: recovery services, workforce training, and employment. A fourth element, systems interventions, was added to capture broader themes. Table 1 displays a summary distribution of ideas. More ideas were identified about recovery support service issues than workforce training or employment issues. Lack of community-located recovery services was consistently cited by people in recovery, families affected by substance abuse, and local support givers such as churches, recovery groups, and law enforcement. Another common challenge was a lack of local coordination among existing services and organizations, resulting in gaps in communication and services coordination. Participants also acknowledged a general lack of focus on employment as a guiding goal for recovery efforts.

The diversity of input assisted in clarifying problems. Personal stories of recovery journeys and deep-felt concerns of family and other community supporters described the impact of personal problems, service gaps, and community issues. Workforce trainers and recovery service providers added depth to themes of assessing and supporting personal readiness for recovery, job training, and finding work. Treatment and recovery service providers combined stories of individual cases and population-level statistics to identify success factors and systemic barriers. Some themes overlapped—lack of transportation, safe and available housing, and meeting social needs like childcare. Participants called for redesigning training systems to focus on the special needs of those in recovery. Employers acknowledged local workforce shortages and the economic need to reengage those in recovery into the job market. Attendees who experienced recovery emphasized that gaining and maintaining meaningful work was a prime facilitator during the recovery process. Ideas emerged for how to encourage greater cooperation between employers and recovery services, including continuity of important medication-assisted therapy services. Those in recovery, advocates, and law enforcement generated ways to overcome legal barriers and restrictive employment practices and regulations.

The most challenging ideas emerged during facilitated discussions between participants focusing on different elements of the Recovery Ecosystem Model. Recovery services and workforce training programs discussed suggestions about how to better coordinate their services through colocation, shared personnel, and peer-to-peer case management. Traditional skills development programs were challenged by employers to include greater soft skills training for clients in recovery, such as time management, work readiness, social skills, and work–family balance. All sectors acknowledged real challenges in promoting community awareness of their own substance-abuse issues that could lead to broad support for individuals in recovery and appreciation of employers that accept perceived risks in hiring those in recovery.

Sorting listening session and public meeting ideas using the planning pyramid format added breadth to data analysis. Long lists of problems were combined to produce rich themes that realistically intertwined individual and systems issues. While fewer in number, a list of goals provided clear statements of what participants felt needed to change. Many local strategies were synthesized into generic themes based on descriptions of similar field-tested practices and policies from different states. Table 2 presents sample ideas combining elements of the Recovery Ecosystem Model with planning pyramid steps. This display demonstrates how ideas generated in different states can be used for local and regional recovery ecosystem model planning.

### ARC Substance Abuse Advisory Council Recommendations

The Council’s multi-sector composition enabled full and rich discussions about both individual and systems problems. Important upstream causal and associated factors identified many different sectors as both part of the problem and potentially part of solutions. The vertical mix of local, regional, and state representatives aided lively debate to clarify perceptions of missions and to define optimal roles within an ecosystem. Representation from all thirteen states helped to define regional similarities and differences and generate a broad overview of characteristics of effective practices, programs, and policies.

Over time ARC has deployed its attention and resources to address regional issues using four general strategies. The Council reviewed each recommendation, using a “Power of Four P’s” outline, to determine which strategies would be appropriate while also ensuring alignment with ARC’s mission. For example, recommendations designed for ARC to introduce the new recovery ecosystem framework including the workforce reentry goal, would gain national and state policy maker attention. This aligns with ARC’s Power of presence in both Washington DC and the thirteen state capitals. Developing new funding and redirecting existing funding to support recovery ecosystems fit ARC’s Power of the Purse. Recommendations that encouraged cooperative alliances with other national and state agencies reinforced ARC’s Power of Partnership strategy. Council recommendations encouraging news reports, web sites, and conferences to introduce and disseminate best practices and results of community interventions illustrate ARC’s Power of the Press. The Four P’s review helped members consider stepped approaches to implement final recommendations. This approach also reinforced ARC’s unique federal–state–local structure as a means to open avenues for true intergovernmental vertical collaboration, coupled with opportunities for multisector horizontal collaboration within communities.

The Council generated a set of common beliefs about the recovery ecosystem. Six declarative sentences formed the rationale for their recommendations. These “We Believe” statements became the preface in the recommendations report:

- Creation of recovery ecosystems is a sustainable solution to the regional substance-abuse epidemic that will benefit many sectors of communities.
- A successful recovery ecosystem will improve workforce participation and significantly contribute to a more viable economy in the Appalachian region.
- The combined understanding and energy of local leaders will lead to tested approaches that meet the unique needs of communities through structured recovery ecosystems that are adaptable across the region.
- The infrastructure for a successful recovery ecosystem should be carefully crafted, deliberately implemented, and consistently evaluated. Lessons from the development of ecosystems should be shared as learning opportunities for all communities.
- Long-term commitments by communities to support recovery to work and by employers to provide competitive compensation are critical.
- Engaging the lived experience of people in recovery is critical to effect change, reduce fragmentation, and improve navigation of services.

The Council’s fourteen recommendations can be found in Table 3. Each full recommendation^15^ includes specific problem statements, goal(s) for change, and recommended strategies drawn both from members’ experience and ideas generated in the listening sessions. Recommendations were not presented in a prioritized list but rather in a general sequential flow, beginning with articulation of the Recovery Ecosystem Model with an evaluated pilot deployment to communities. Several recommendations focused on improving linkages between recovery and training organizations. In general, three recommendations addressed recovery ecosystems model development and testing (#1, 4, and 8), four addressed educational approaches in support of recovery ecosystems development and operation (#2, 3, 5, and 11), four addressed actions to promote recovery ecosystem performance (#6, 7, 12, and 14), and three addressed sector-specific actions to enhance potential effectiveness (#9, 10, and 13).

## IMPLICATIONS

The four-step process was successful in gaining the wisdom of the field by hearing voices of the recovery community, workforce programs, and employers as part of ARC’s response to the recovery-to-work issue. The entire process— including the listening sessions and public meetings, the Advisory Board deliberations, and full Commission review and approval—consumed less than 12 months. The process generated a credible justification for ARC to pursue investments in the niche of recovery-to-work initiatives. Extensive involvement of state and local interests in organizing meetings and recruiting participants and speakers was instrumental in quickly obtaining input that was integrated into the 2020 ARC grant offerings.

The listening sessions were not conducted in all thirteen states of the region; therefore, the input may not represent ideas from across the entire Appalachian Region. However, this process confirms and expands similar findings from other ARC substance-abuse and labor workforce participation studies that encompass the entire region. Potential cultural biases of the findings were addressed through inclusion of a broad set of participants and sectors with diversified interests. The interpretation-of-findings process followed the principles of community-based participatory research throughout.^16^

Agreement was found on fundamental principles. If Appalachia is not to be left behind in the national economic upswing experienced prior to the COVID-19 pandemic,^17^ substance-abuse recovery is an important component of broad regional workforce availability and improvement strategies. There is no single government agency charged with this task, nor does any governmental or nongovernmental organization have the capacity to address it, nor is any single program practice ready to be disseminated across communities. The ARC Recovery Ecosystem Model is helpful to understand the complexity of service systems issues and individual challenges of those who are in recovery. There is a need to define more fully “required recovery ecosystem elements,” including organizational linkages and operational protocols, that help prevent those in recovery from falling through service system cracks. Established ecosystems are seen to be an important precursor for effective partnerships with state and federal substance-abuse and workforce funding opportunities.

## Figures and Tables

**Table 1:** Percentage of Ideas from Listening Sessions and Public Meetings by Recovery Ecosystem Elements and Planning Pyramid Steps

Recovery Ecosystem Model Elements	Planning Pyramid Steps
57% Recovery Support Services	16% Problem Statements
17% Workforce Training	12% Goals for Change
26% Employment	58% Alternative Strategies
	14% Best Practice Programs and Policies

**Table 2:** Matrix of Ideas Combining the Recovery Ecosystem Model and Planning Pyramid

	Recovery services	Workforce	Employment	Broad Systems Interventions
**Problem statements**	There is not a strong set of talking points about how to eliminate stigma of addiction and medically assisted therapy. There is no voice for long-term recovery because of anonymity issues. A marketing plan needed.	Training programs are not prepared to address the multiple complex challenges faced by those in recovery that negatively impact their ability to join and willingness to maintain enrollment.	Judicial system guidelines and records create long term employment barriers for the large percentage of non-violent convicted felon many of whom have substance use disorders.	There is a lack of connectedness among federal, state, and local resources and services which does not act to effectively network government and non-profits to address needs and gaps in a systematic way.
**Goals for change**	The system of services for clients will eliminate gaps by creating community-specific hubs where human services professionals without bias and stigma help begin the “recovery to life to work process”	Begin to see those in recovery as assets (prospective employees) rather than liabilities in a community and to society	Campaigns will be developed to promote recovery friendly workplaces (similar to veteran-friendly workplaces) with statewide convenings to highlight, award, and incentivize HR directors	Funding will require data sharing to identify service system gaps and clarify individuals’ needs to be addressed to improve success in recovery. Future funding will target addressing gaps in programs.
**Alternative strategies to achieve goals**	Give everyone a personal reintegration specialist contact who manages whole range of services: transportation and drivers’ licenses, transitional housing, court costs, adult education programs, access to legal services, medical care, and mental health care, ready to work programs, and social workers services	Identify and promote linkages between recovery services and workforce training agencies including: contracts for services; jointly operated programs; workforce partnership meetings; cross-sector training; and grants to test and demonstrate new approaches.	Employers interested in hiring and retaining people in recovery through human resources department training, addressing job safety/security, OSHA, hiring regulations, workers comp issues and culture change.	Design a process and framework for communities to develop community-based multi-sector task forces to assess local problems, conduct asset mapping, and develop recovery ecosystem plans.
**Effective programs**	Models of jail and prison pre-release handoffs to treatment and community recovery initiatives that offer mental health services, medication assistance and therapies, linked to job skills training with peer supports.	Use individualized workforce training and employment readiness assessment tools and evaluation processes to develop short-term training and employment readiness plans to match interests and address potential work challenges.	Conduct training for employers on EAP legalities, policies templates for hiring, and operational issues for maintaining a supportive work environment for employees in recovery.	ARC funding should promote evaluation and measurement of recovery ecosystems and develop toolkits that share best practices.

**Table 3:** ARC Substance Abuse Advisory Council Recommendations, 2019

1. Develop a recovery ecosystem model that addresses stakeholder roles and responsibilities as part of a collaborative process that develops infrastructure and operations, and ARC should fund deployment of local planning and implementation of the model, and examine funding models to sustain the recovery ecosystem.
2. Develop and disseminate a playbook of solutions for communities addressing common ecosystems gaps and services barriers.
3. Convene regional leaders to educate them about the disease of addiction, encourage their engagement in the recovery ecosystem development process, and use resource clearinghouses, playbooks, toolkits, and other products. Formation of partnerships should be a primary objective of the convening process.
4. Fund community pilot projects to demonstrate strategies that address common Appalachian recovery to work issues that negatively impact regional workforce and employment gaps.
5. Support communities to create and sustain clearinghouses, both physical and virtual, that include federal, state, and local resources to guide those seeking help for persons in active addiction, or those in recovery and seeking meaningful employment.
6. Identify one to three commonly available performance metrics for each step of the recovery ecosystem model, including tools and data collection processes for each step of the model, to measure ecosystem effectiveness and capture progress made by individuals in recovery. The measures should be commonly available and reflect the needs and concerns of different stakeholders.
7. Develop and disseminate a model individualized workforce training and employment readiness assessment and evaluation process that helps persons in recovery to secure gainful employment that is meaningful to the individual and allows them to support themselves financially.
8. Develop model workforce training programs that incorporate recovery services with appropriate evaluation measures.
9. Research and identify social program eligibility and restrictions that may discourage participants from seeking employment.
10. Create, publish, and disseminate a report which inventories and maps effective best practices in legal deflection and diversion programs as well as state programs that incentivize hiring of persons in recovery with criminal records related to drug charges across the Appalachian region.
11. Convene experts to develop and disseminate an employer best practices toolkit to educate employers and human resource experts in recruiting, selecting, managing, and retaining employees who are in recovery.
12. Fund local liaison positions across Appalachia responsible for promoting a recovery ecosystem by building bridges between employers, workforce development agencies, and recovery organizations, and disseminating an employer best practices toolkit.
13. Fund development of Collegiate Recovery Programs (CRPs) in Appalachian technical schools, small colleges, and universities designed to establish and nurture authentic student-centered communities that focus on interests, wellness, and success for students seeking and living in recovery.
14. Convene a meeting of interested stakeholders to identify how transportation barriers negatively impact recovery to work efforts in rural communities and regional workforce participation, and profile innovative partnerships and funding models that lead to sustainable community solutions enabling individuals to stay in recovery, training programs, and employment.
